# Identification and relationship of quality of life and self-care ability among Chinese patients with traumatic spinal cord injuries: a cross-sectional analysis

**DOI:** 10.1590/1414-431X2021e11530

**Published:** 2021-10-29

**Authors:** Li Jiang, Li Sun, Qingtao Meng

**Affiliations:** 1 Department of Nursing, Dalian Third People's Hospital, Dalian, China; 2 Department of Nursing, the First Affiliated Hospital of Dalian Medical University, Dalian, China; 3 Spinal Trauma Ward, Dalian Third People's Hospital, Dalian, China

**Keywords:** Modified Barthel Index, Paraplegia, Pressure injury, Quality of life, Spinal cord injuries, Tetraplegia

## Abstract

Improving the quality of life of patients with complete spinal cord injuries is an urgent objective of the Chinese Department of Health. For better management of spinal cord injuries, it is necessary to understand the background of the patients. A total of 392 patients aged ≥18 years with traumatic spinal cord injuries (≥1 year of history) were attending the rehabilitation center of the Institutes. A total of 7 (2%) patients reported low quality of life, 200 (51%) patients reported moderate quality of life, 181 (46%) patients reported good quality of life, and 4 (1%) patients reported excellent quality of life. Male patients (P=0.042), patients with college or more education (P=0.039), incomplete spinal cord injuries (P=0.045), paraplegia (P=0.046), and absence of pressure injury (P=0.047) were associated with higher quality of life. A total of 81 (21%) patients were dependent on the caregiver, 85 (22%) patients were highly dependent on the caregiver, 155 (40%) patients were moderately dependent on the caregiver, 60 (15%) patients were mildly dependent on the caregiver, and 11 (2%) patients were independent for activities of daily living. An incomplete spinal cord injury (P=0.045) and paraplegia (P=0.041) were associated with higher independence in activities of daily living of patients. The independence in activities of daily living and quality of life of the Chinese population with complete spinal cord injury and tetraplegia are poor (Level of Evidence: IV; Technical Efficacy Stage: 5).

## Introduction

Spinal cord injury is one of the leading health problems and affects the overall quality of life of the affected patients (1). It has serious effects on the lives of individuals, their families, and society (2) by causing serious disabilities of the patient (3). Traumatic spinal cord injuries are a lifelong problem and require care to decrease complications (4). According to the 2013 report of the World Health Organization, the incidence of spinal cord injury is 40 to 80/million population/year (5). There are more than one million patients with traumatic brain injuries who are impaired in daily life activities and this number is increasing by 10% per year in mainland China (6). Patients with complete spinal cord injuries have slower recovery than those with incomplete spinal cord injuries (7).

Many complications are associated with spinal cord injury, such as loss of motor activity and sensations (8,9), respiratory and cardiovascular complications, urinary complications, bowel complications, possibilities of vein thrombosis, pressure ulcers, edema, and pain symptoms (10). Also, after spinal cord injury, patients are impaired in daily activities, have a poor quality of life, and are dependent on caregivers (8,10). Psychological distress and poor mental health are associated with spinal cord injury (11). Therefore, patients need multidisciplinary rehabilitation intervention(s) to overcome caregiver dependence in their daily activities (12). Demographic, clinical, and socioeconomic factors affect caregiver dependence for the daily routine (13).

Improving the quality of life of patients with complete spinal cord injuries is an urgent objective of the Department of Health of China because community participation of these patients is poor in mainland China (6). Also, patient information is important for improving independence in daily activities (14). For the best management of spinal cord injuries, it is necessary to understand self-care abilities and quality of life of individuals and the relationship to demographic, clinical, and socioeconomic factors (2). Rehabilitation can decrease dependency even in the highest level of injury. Better health-related conditions and financial aspects improve the quality of life of patients with spinal cord injuries (15).

The objective of this cross-sectional retrospective analysis was to evaluate the self-care ability and quality of life of Chinese patients with traumatic spinal cord injuries. Also, the relationship between demographic, clinical, and socioeconomic parameters, spinal cord injury-related factors, self-care ability, and quality of life of patients was assessed.

## Material and Methods

### Ethics approval and consent to participate

The study protocol (FDMU1521 dated 17 February 2021) was approved by the First Affiliated Hospital of Dalian Medical University Review Board and the Chinese Nursing Association. The study adhered to the law of China and the 2008 Declaration of Helsinki. Being a retrospective study, the registration in the Chinese trial registry was waived by the institutional review board.

### Inclusion and exclusion criteria

Patients aged 18 years or more with traumatic spinal cord injuries (more than 1 year of history) due to accident, fall, or bullet injuries and available at the rehabilitation center of the institutes were included in the analysis.

Patients with traumatic spinal cord injuries and on treatment for psychiatric problems were excluded from the study because such treatment may affect the quality of life of patients.

### Sample size calculations

For 13 parameters on self-care ability and quality of life among patients with traumatic spinal cord injuries, 5% two-sided type-I error (α=0.05), 80% power (β=0.2), and 95% confidence interval, a minimum of 130 patients was required (sample size) (1).

### Outcome measures

Data regarding age, gender, marital status, educational status, duration of spinal cord injuries, and social and economic status were retrospectively collected from the patients' records. These data were stored in the hospital records as part of the daily routine and quality management.

### Spinal cord injury-related factors

#### Neuropathic pain

A numeric rating scale was used for the measurement of neuropathic pain intensity. The score range is 0 to 10: 0 indicates no pain, 1-3 indicates mild pain, 4-6 indicates moderate pain, 7-9 indicates severe pain, and 10 indicates worst possible pain (16).

#### Pressure injury of spinal cord injury

This was determined by physical examinations according to the National Pressure Ulcer Advisory Panel (17).

#### Level of spinal cord injury

Level of injury was classified as tetraplegia or quadriplegia (spinal cord injuries at cervical segment and patients have weakness of upper limbs), paraplegia (spinal cord injuries at the thoracic, lumbar, or sacral segment, and patients have weakness of lower limbs), complete injury (all motor and sensory functions are absent), and incomplete injury (partial motor and sensory functions are retained). The level of spinal cord injuries was classified according to the institutional protocol.

### Quality of life

The mainland Chinese version of the quality-of-life index was used for the evaluation of the quality of life of patients. It includes a total of 28 items (two items of the overall perception of patients regarding quality of life of patients, seven items of physical health, six items of physiological well-being, three items of social relationships, eight items of environment, and two unique items of China). Each item is rated on a 5-point Likert scale (1 to 5) indicating the extent to which the item affects the quality of life: 1 indicates no effect, 2 indicates a little, 3 indicates moderate, 4 indicates good, and 5 indicates excellent. Cronbach's α was 0.952. The quality-of-life index score range is 0-100. The total score is categorized as 0-20: no quality of life; 21-40: little quality of life; 41-60: moderate quality of life; 61-80: good quality of life; 81-100: excellent quality of life (6).

### Activities of daily living

A total of 10 activities were evaluated using the modified Barthel Index score. Activities of daily living were divided into five sub-categories as per need of help of caregiver(s): total dependency (score: 0), substantial help (score: 1-3), moderate help (score: 4-6), minimal help (score: 7-9), and independent (score: 10). The total score is 100 (10×10) and it is categorized as 0-24: total dependency on a caregiver; 25-49: high dependency; 50-74: moderate dependency; 75-94: mild dependency; 95 or more: total independency (18).

### Statistical analysis

SPSS 26.0 (IBM Corp. USA) was used for statistical analyses. A multiple linear regression analysis was performed to examine associations of demographic, clinical, and socioeconomic parameters and spinal cord injury-related factors with the quality of life of patients. Also, the association of those factors with the independence of daily living of patients was examined. All results were considered significant if P<0.05.

## Results

### Study population

**Figure 1:**
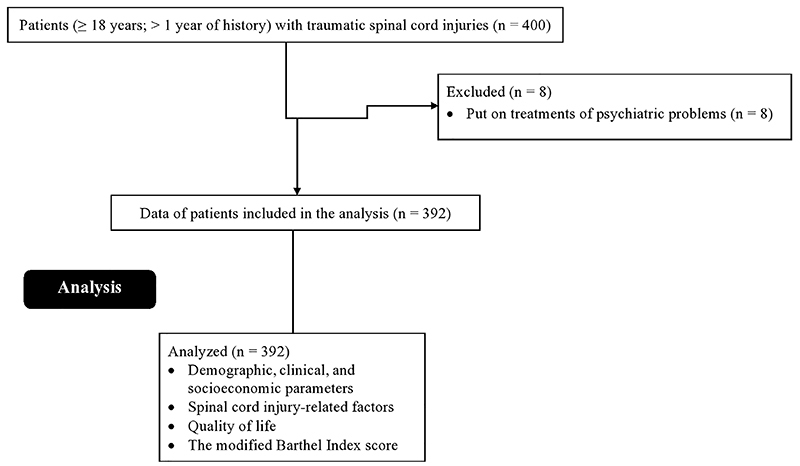
The flow diagram of the study.

From June 15, 2019 to February 11, 2020, data from 400 patients aged 18 years or more with traumatic spinal cord injuries (more than 1 year of history) were available at the rehabilitation center of the Dalian Third People's Hospital and the First Affiliated Hospital of Dalian Medical University. Among them, eight patients were under treatments for psychiatric problems and were excluded from the analysis. Data from 392 patients were analyzed and the flow diagram of the study is shown in Figure 1.

### Demographic, clinical, and socioeconomic data

Patients' age range was 31 to 68 years (48.15±11.15) and mean income was 1,115±302 ¥/month/patient. Most patients had spinal cord injuries due to accidents. The demographic, clinical, and socioeconomic parameters and spinal cord injury-related factors of the enrolled patients are reported in Table 1.

**Table 1:** Demographic, clinical, and socioeconomic parameters and spinal cord injury-related factors of the enrolled patients.

Parameters	Data
Patients enrolled (n)	392
Age (years)	
Minimum	31
Maximum	68
Mean±SD	48.15±11.15
Gender	
Female	115 (29)
Male	277 (71)
Marital status	
Married	250 (64)
Single	142 (36)
Ethnicity	
Han Chinese	356 (91)
Mongolian	30 (7)
Tibetan	4 (1)
Uighur Muslim	2 (1)
Educational status	
High school or less	302 (77)
College or more	90 (23)
Cause of injury	
Accident	215 (55)
Fall	132 (34)
Bullet injuries	45 (11)
Living	
With family	367 (94)
Without family	25 (6)
Duration of spinal cord injuries (years)	
1-5	155 (40)
6-10	102 (26)
11-14	81 (21)
≥15	54 (13)
Classification of injuries	
Complete	279 (71)
Incomplete	113 (29)
Level of injuries	
Tetraplegia or quadriplegia	83 (21)
Paraplegia	309 (79)
Caregiver status	
With caregiver	320 (82)
Without caregiver	72 (18)
Pressure injury	
Presence	327 (83)
Absence	65 (17)
Neuropathic pain	
Minimum	0
Maximum	10
Mean±SD	4.15±1.14

Categorical data are reported as frequency (percentage) and continuous data are reported as means±SD.

### Quality of life

The mean quality-of-life index score was 60±15. Seven (2%) patients reported little quality of life (score: 21-40), 200 (51%) patients reported moderate quality of life (score: 41-60), 181 (46%) patients reported good quality of life (score: 61-80), and 4 (1%) patients reported excellent quality of life (score: 81-100). Male patients (P=0.042), patients with college or more education (P=0.039), incomplete spinal injuries (P=0.045), paraplegia (P=0.046), and absence of pressure injury (P=0.047) were associated with higher quality of life. The associations of demographic, clinical, and socioeconomic parameters and spinal cord injury-related factors with the quality of life of patients are reported in Table 2.

**Table 2:** Multiple linear regression analysis for the association of demographic, clinical, and socioeconomic parameters and spinal cord injury-related factors with quality of life of the enrolled patients.

Parameters	Odds ratio	95% Confidence interval	P-value
Age (≤50 *vs* >50 years)	0.652	0.566-0.762	0.081
Gender (male* *vs* female)	1.112	0.892-1.482	0.042
Marital status (married *vs* unmarried)	0.813	0.516-0.911	0.062
Ethnicity (Han Chinese *vs* others)	0.724	0.561-0.751	0.071
Educational status (college or more* *vs* high school or less)	1.156	0.492-1.392	0.039
Cause of injury (accident *vs* other)	0.821	0.521-0.981	0.069
Living (with family *vs* without family)	0.893	0.551-0.972	0.068
Duration of spinal cord injuries (≤10 *vs* >10 years)	0.912	0.492-0.982	0.067
Classification of injuries (incomplete* *vs* complete)	1.051	0.481-1.512	0.045
Level of injuries (paraplegia* *vs* tetraplegia or quadriplegia)	1.142	0.452-1.625	0.046
Caregiver status (presence *vs* absence)	0.763	0.551-0.862	0.056
Pressure injury (absence* *vs* presence)	1.044	0.762-1.211	0.047
Neuropathic pain (absence *vs* presence)	0.823	0.512-0.992	0.062

An odds ratio of more than 1 with P<0.05 was considered significant. *Significant parameter for a higher quality of life.

### Activities of daily living

A total of 81 (21%) patients were dependent on the caregiver (modified Barthel Index score: 0-24), 85 (22%) patients were highly dependent on the caregiver (score: 25-49), 155 (40%) patients were moderately dependent on the caregiver (score: 50-74), 60 (15%) patients were mildly dependent on the caregiver (score: 75-94), and 11 (2%) patients were independent (score ≥95) for activities of daily living (Table 3). An incomplete spinal cord injury (P=0.045) and paraplegia (P=0.041) were associated with higher independence in activities of daily living. The associations of demographic, clinical, and socioeconomic parameters and spinal cord injury-related factors with activities of daily living dependence are reported in Table 4.

**Table 3:** Activities of daily living of patients with traumatic spinal cord injuries.

Activities	Modified Barthel Index score				
	Total dependency	Substantial help	Moderate help	Minimal help	Independent
	0	1-3	4-6	7-9	10
Toilet	5 (1)	57 (14)	152 (39)	148 (38)	30 (8)
Care of bladder	35 (9)	75 (19)	115 (29)	141 (36)	26 (7)
Bowels	25 (6)	71 (18)	131 (34)	121 (31)	44 (11)
Ambulation	67 (17)	77 (19)	109 (28)	121 (31)	18 (5)
Feeding	15 (4)	81 (21)	131 (33)	161 (41)	4 (1)
Bathing	17 (4)	88 (22)	189 (49)	91 (23)	7 (2)
Dressing	22 (6)	88 (22)	87 (22)	184 (47)	11 (3)
Grooming	81 (21)	75 (19)	131 (34)	96 (24)	9 (2)
Stair climbing	95 (24)	131 (34)	142 (36)	18 (5)	6 (1)
Transfers	115 (29)	117 (30)	121 (31)	25 (6)	14 (4)

Data are reported as frequency (percentage).

**Table 4:** Multiple linear regression analysis of the association of demographic, clinical, and socioeconomic parameters and spinal cord injury-related factors with activities of daily living of patients.

Parameters	Odds ratio	95% Confidence interval	P-value
Age (≤50 *vs* >50 years)	0.712	0.681-0.812	0.632
Gender (male *vs* female)	0.811	0.562-0.891	0.561
Marital status (married *vs* unmarried)	0.651	0.592-0.792	0.651
Ethnicity (Han Chinese *vs* others)	0.722	0.612-0.852	0.612
Educational status (college or more *vs* high school or less)	0.731	0.667-0.842	0.623
Cause of injury (accident *vs* other)	0.651	0.591-0.801	0.669
Living (with family *vs* without family)	0.669	0.582-0.799	0.667
Duration of spinal cord injuries (≤10 *vs* >10 years)	0.711	0.671-0.812	0.623
Classification of injuries (incomplete* *vs* complete)	1.051	0.762-1.372	0.045
Level of injuries (paraplegia* *vs* tetraplegia or quadriplegia)	1.091	0.752-1.481	0.041
Caregiver status (presence *vs* absence)	0.652	0.561-0.821	0.641
Pressure injury (absence *vs* presence)	0.664	0.552-0.812	0.669
Neuropathic pain (absence *vs* presence)	0.658	0.591-0.809	0.692

An odds ratio of more than 1 with P<0.05 was considered significant. *Significant parameter for higher independence in activities of daily living.

## Discussion

The current study found that more than half of the enrolled patients were dependent on caregivers for activities of daily living after traumatic spinal cord injuries. The results are consistent with that of the retrospective study on the Bangladeshi population with traumatic and non-traumatic spinal cord injuries (19) and a cross-sectional study on the Jordan population with traumatic spinal cord injuries (1). Spinal cord injuries may make the patient dependent on caregivers. The dependency from the caregiver is different if participants are in rehabilitation or at home. In the current study, the enrolled patients were at home or discharged from rehabilitation. Therefore, more than half of the enrolled patients were dependent on caregivers for activities of daily living.

The current study found that patients who had an incomplete spinal injury and paraplegia have more independence for activities of daily living. The results were in accordance with a cross-sectional study on the Jordan population with traumatic spinal cord injuries (1), a cross-sectional survey on the North American population with traumatic spinal cord injuries (13), and a cross-sectional study on the Korean population (20). Patients with incomplete spinal injuries have partial impairment of motor and sensory functions (21). Therefore, patients with incomplete spinal injuries can perform some movements independently. Also, patients with paraplegia have weakness of lower limbs, and patients can perform movements of upper extremities (1). Patients with incomplete spinal cord injuries and paraplegia may have higher independence for activities of daily living.

The study found that only 47% of patients had a good quality of life or higher. The results agree with those of cross-sectional studies on the Jordan population (1), Iranian population (22), and Chinese population (6) with traumatic spinal cord injuries. Traumatic spinal cord injuries affect the essential elements of daily living (1). Individuals with traumatic brain injuries have to take part in community activities because the problem is permanent (4). Also, patients with traumatic spinal cord injuries live more frequently alone, which leads to poor mental health (11).

A higher quality of life of patients was associated with male gender, patients with college or more education, incomplete spinal injuries, paraplegia, and absence of pressure injury. Patients with incomplete spinal cord injuries and paraplegia had higher independence in activities of daily living, which results in a higher quality of life. Patients with pressure injuries have limited physical and social activity, which leads to poor quality of life. Patients with higher education level have more awareness and ability to adopt a healthy life style, which leads to a higher quality of life (1). Also, female patients have cultural issues in the Chinese society, which leads to poor quality of life (6). The findings of the current study agree with those of cross-sectional studies on the Jordan population (1), Iranian population (22), and Chinese population (6) with traumatic spinal cord injuries and with those of longitudinal multicenter studies on the European population (15,23). The strengths and weaknesses of patients after spinal cord injuries must be taken into account to improve the quality of life of patients.

As limitations, this study did not assess caregiver data, such as educational level, age, and gender, which may affect the quality of life of patients (1). People living in developed cities in mainland China have better rehabilitation facilities than those of small cities and towns (6). Therefore, the generalizability of the results to the Chinese population is impeded.

### Conclusions

This study analyzed self-reported quality of life questionnaires of a spinal cord injury sample. Complete spinal cord injury and tetraplegia or quadriplegia can make the patient dependent on caregivers. Traumatic spinal cord injuries had adverse effects on the quality of life of patients. The activities of daily living and quality of life of the Chinese population with traumatic spinal cord injuries were poor except for patients with incomplete spinal cord injuries and paraplegia. Providing adequate nursing of patients may improve their activities of daily living and quality of life.
